#  Risk factors and trajectories of self-harm, neurodevelopmental disorders, and mental health conditions in pupils Educated in Other Than At School (EOTAS) in Wales: a retrospective nationwide electronic cohort study 

**DOI:** 10.23889/ijpds.v9i5.2769

**Published:** 2024-09-18

**Authors:** Olivier Y. Rouquette, Marcos del Pozo Banos, Sze Chim Lee, Ann John

**Affiliations:** 1 Swansea University Medical School

## Objective and Approach

Pupils ‘Educated in Other Than At School’ (EOTAS) are some of the most vulnerable learners, who, for reasons such as mental health or behavioural difficulties, do not attend a mainstream school. We linked population data from Education Wales (EDUW) between 2010-19 to primary and secondary healthcare records. Individuals included in the EOTAS dataset aged from 7 to 18 years were identified as cases. Controls were pseudo-randomly selected based on equivalent age distribution. We used regression approach to compare characteristics of EOTAS pupils with controls and evaluate their risks of self-harm and mental health conditions before and after being in EOTAS provision.

## Results

Being male, high deprivation levels, and previous records childhood maltreatment, self-harm, and mental health conditions resulted in higher odds of being in EOTAS provision. Female in EOTAS provision entered at a later age, and with higher proportion of co-morbidities. Pupils in EOTAS provision had increased incidence of self-harm and mental health conditions from one year after entering EOTAS provision up to 24 years of age than controls with similar characteristics.

## Conclusions

An important proportion of pupils in EOTAS provision are from the most deprived areas in Wales, with complex life trajectories, social, emotional, and mental health needs.

## Implications

EOTAS provision is not sufficient to attend the social, emotional, and mental health needs of their pupils. Long-term support and a better integration with social care, primary and specialist mental health services is required.

